# Expression analysis of glutathione S-transferases and ferritins during the embryogenesis of the tick *Haemaphysalis longicornis*

**DOI:** 10.1016/j.heliyon.2020.e03644

**Published:** 2020-03-30

**Authors:** Emmanuel Pacia Hernandez, Kei Shimazaki, Hiroko Niihara, Rika Umemiya-Shirafuji, Kozo Fujisaki, Tetsuya Tanaka

**Affiliations:** a Laboratory of Infectious Diseases, Joint Faculty of Veterinary Medicine, Kagoshima University, 1-21-24 Korimoto, Kagoshima 890-0056, Japan; b Department of Pathological and Preventive Veterinary Science, The United Graduate School of Veterinary Science, Yamaguchi University, Yoshida, Yamaguchi 753-8515, Japan; c National Research Center for Protozoan Diseases, Obihiro University of Agriculture and Veterinary Medicine, Obihiro, Hokkaido 080-8555, Japan; d National Agricultural and Food Research Organization, 3-1-5 Kannondai, Tsukuba, Ibaraki 305-0856, Japan

**Keywords:** Developmental biology, Genomics, Transcriptome, Proteomics, Oxidative stress, Embryology, Veterinary medicine

## Abstract

In the tick life cycle, embryogenesis is the only stage of development wherein no blood meal is required. Nevertheless, even in the absence of a blood meal, which is the source of nutrients as well as the ferrous iron and heme that could cause oxidative stress in ticks, malondialdehyde (MDA) has been reported to increase during this period. Additionally, the knockdown of some oxidative stress–related molecules such as ferritin has resulted in abnormal eggs and embryonic death.

Here, we investigate the gene and protein expression profiles of the identified glutathione S-transferases (GSTs) and ferritins (Fers) of the tick *H. longicornis* during embryogenesis through quantitative reverse transcription polymerase chain reaction (RT-qPCR) and Western blotting, respectively. We also confirm the lipid peroxidation and ferrous iron concentration level using a thiobarbituric acid reactive substances (TBARS) assay. Finally, we attempt to correlate these findings with the events occurring by establishing a staging process in *H. longicornis* embryos.

Lipid peroxidation increased during the course of embryogenesis, as does the amount of GST proteins. On the other hand, the *GST* genes have high expression at the 1^st^ day post-oviposition, during the early stage of embryogenesis and at day 10 during the period wherein the germ band is observable. *Fer* gene expression also starts to increase at day 10 and peaks at day 15. In the ferritin proteins, only the secretory ferritin (Fer2) is detected and constitutively expressed during embryogenesis.

Events occurring during embryogenesis, such as energy production and iron metabolism for cellular proliferation and differentiation cause oxidative stress in the embryo. To counteract oxidative stress, it is possible that the embryo may utilize oxidative stress–related molecules such as GSTs and Fer2, which could be either maternally or embryo-derived.

## 1 Introduction

The hard tick *Haemaphysalis longicornis* has been gaining attention recently because it is a vector of several disease-causing pathogens. These ticks, originally known to be native to Eastern Asia and to have been established in Australia and New Zealand, are now also reported in various places in North America and Pakistan (Sonenshine and Roe, 2014; Raghavan et al., 2019; Ali et al., 2019). They are obligate blood-feeding arthropods that need to feed on blood in almost all developmental stages, except during embryonic and larval development. Embryogenesis is a crucial stage in the life cycle of these ticks (Zhang et al., 2019). Due to the lack of blood during this crucial process, the egg mainly relies on the yolk to furnish it with the energy and nutrition required for development (Martins et al., 2018).

Glutathione S-transferases (GSTs) are enzymes that act in the excretion of physiologic and xenobiotic substances, protecting cells against chemical toxicity and stress. Aside from this, they are involved in the catalysis of products of oxidative stress (Hayes and Pulford, 1995). A previous study revealed that GST activities increase during embryonic and larval development, but the exact GST molecule contributing to this increase remains unknown (Freitas et al., 2007). In *H. longicornis*, two GST molecules (GST1 and GST2) have already been identified and characterized during blood feeding and acaricide exposure, but their role during embryonic development is yet to be determined (da Silva Vaz et al., 2004; Hernandez et al., 2018a, 2018b).

Iron molecules are important in many metabolic processes, including energy metabolism and DNA replication. They are important in the embryogenesis of mammals and may also be applicable to other species (Hentze et al., 2004). However, the Fe^2+^ form has been shown to catalyze the Fenton reaction to produce reactive oxygen species (ROS), which damage cells (Whiten et al., 2017). Ferritin in ticks has an intracellular type (Fer1) and a secretory type (Fer2) (Hajdusek et al., 2009; Galay et al., 2013, 2014b). Fer1 has the function of retaining Fe^3+^ transformed from the toxic Fe^2+^ as an antioxidant molecule; conversely, Fer2 transports Fe^3+^ from the midgut to the ovary, suggesting that it plays an important role as an antioxidant and in embryogenesis (Galay et al., 2015). Furthermore, silencing the *Fer2* gene has been shown to cause a decreased hatching rate and the formation of abnormal eggs (Galay et al., 2013).

Antioxidant molecules are currently being explored as target molecules for tick control. Aside from these, molecules of embryonic origin are also looked upon as potential targets. Previous studies have shown that malondialdehyde (MDA), which is an index of lipid peroxidation and oxidative stress, has an increased expression during embryonic development (Freitas et al., 2007). This could be an indication that during embryonic development, oxidative stress occurs in the tick that could result in lipid peroxidation. The occurrence of oxidative stress may be related to the acquired maternal iron. In this study, we attempted to identify the possible roles of the antioxidant molecules GST and Fers during embryonic development by analyzing their expression and correlating their possible functions during the various events of embryonic development.

## 2 Materials and methods

### 2.1 Ticks and experimental animals

In this study, the parthenogenetic Okayama strain of *H. longicornis* was used in all experiments. Ticks were maintained for several generations at the Laboratory of Infectious Diseases, Joint Faculty of Veterinary Medicine, Kagoshima University, Kagoshima, Japan, by feeding on the ears of Japanese white rabbits (KBT Oriental, Saga, Japan) (Fujisaki, 1978). Rabbits were also used in all tick infestation experiments. Animals were kept at 25 °C and 40% relative humidity, with a constant supply of water and commercial feed. The ticks were maintained in glass tubes sealed with a cotton plug and kept at 15 °C in an incubator until use. Engorged female ticks were collected and placed on 24-well plates and placed in a humid chamber in a 25 °C incubator. During egg-laying, the eggs were collected daily for at least one week, placed in a separate 24-well plate and kept in the same incubator. The eggs were then collected every 5 days up to the 20th day post-oviposition. The care and use of experimental animals in this study were approved by the Animal Care and Use Committee of Kagoshima University (approval number VM15055) in accordance with the standards of the Association for Assessment and Accreditation of Laboratory Animal Care (AAALAC) International.

### 2.2 Total RNA extraction and quantitative reverse transcription PCR (RT-qPCR) analysis

Total RNA was extracted from eggs on different days during oviposition (days 0, 5, 10, 15, and 20) (Freitas et al., 2007). Egg samples were homogenized using an automill (Tokken, Chiba, Japan) and were added to TRI Reagent® (Molecular Research Center, OH, USA). RNA extraction was performed following the manufacturer's protocol. Subsequently, single-strand cDNA was prepared by reverse transcription using the ReverTra Ace® qPCR RT Master Mix (Toyobo, Osaka, Japan), following the manufacturer's protocol. Transcription analysis of *GST1*, *GST2*, *Fer1*, and *Fer2* genes was performed through RT-qPCR using THUNDERBIRD™ SYBR® qPCR Mix (Toyobo) with an Applied Biosystems 7300 Real-Time PCR System using gene-specific primers, GST1 real-time forward and GST1 real-time reverse primers, GST2 real-time forward and GST2 real-time reverse primers, Fer1 real-time forward and Fer1 real-time reverse primers, and Fer2 real-time forward and Fer2 real-time reverse primers (Table 1). Standard curves were made from fourfold serial dilutions of cDNA of the whole body of adult female ticks fed for 3 days. The PCR cycle profile was as follows: 95 °C for 10 min, 40 cycles of a denaturation step at 95 °C for 15 s, and an annealing/extension step at 60 °C for 60 s. The data was analyzed using Applied Biosystems 7300 system SDS software. In the first step of RT-qPCR, *actin*, *tubulin*, *P0*, and *L23* genes were evaluated for standardization (Nijhof et al., 2009; Hernandez et al., 2018b). *P0* gene was selected as an internal control.

**Table 1:** Gene-specific primers used in this study.

Primer	Sequence (5' → 3′)
GST1 real-time forward	CTTCTTGGATCTTGGCGGGT
GST1 real-time reverse	CGATGTCCCAGTAGCCGAG
GST2 real-time forward	CCCTTCCGGGAATGAAGGAG
GST2 real-time reverse	GATCGCTCAGCAGTCGTCAG
Fer1 real-time forward	ATGGCCGCTACTCAACCCCG
Fer1 real-time reverse	GAACTTGTGGAAGCCCGGCA
Fer2 real-time forward	ATGCTCCCGATCCTGATCTT
Fer2 real-time reverse	GGCCATCTGCATGTAGACCAA
P0 real-time forward	CTCCATTGTCAACGGTCTCA
P0 real-time reverse	TCAGCCTCCTTGAAGGTGAT
L23 real-time forward	CACACTCGTGTTCATCGTCC
L23 real-time reverse	ATGAGTGTGTTCACGTTGGC
Actin real-time forward	ATCCTGCGTCTCGACTTGG
Actin real-time reverse	GCCGTGGTGGTGAAAGAGTAG

### 2.3 Protein extraction and Western blot analysis

Protein was extracted from eggs on different days post-oviposition. Egg samples were homogenized using an automill (Tokken) and then suspended in PBS treated with Complete Mini Proteinase Inhibitor Cocktail Tablets (Roche, Mannheim, Germany). The egg samples were bath sonicated for 5 min, amplitude 45 (AS ONE, Osaka, Japan), and then centrifuged at 20,600 × *g* at 4 °C for 5 min, wherein the supernatant was recovered. After sonication and recovery of the supernatant, the egg protein concentration was measured using a Micro BCA kit (Thermo Scientific, Rockford, IL, USA). Equal protein concentrations were separated with 12% SDS-polyacrylamide gel electrophoresis (SDS-PAGE) and transferred to a polyvinylidene difluoride (PVDF) membrane (Millipore, Billerica, MA, USA). The membrane was blocked at 4 °C overnight with 3% skim milk in PBS containing 0.05% Tween 20 and then incubated with a primary antibody using mouse anti-GST1, -GST2, -Fer1, or -Fer2 serum (1:1000 dilution) at 37 °C for 1 h. After incubation with horseradish peroxidase–conjugated goat anti-mouse IgG (1:50,000 dilution; DakoCytomation, Glostrup, Denmark) at 37 °C for 1 h, the signal was detected using Clarity™ Western ECL Substrate (Bio-Rad Laboratories, Hercules, CA, USA). It was analyzed using FluorChem FC2 software (Alpha Innotech, San Leandro, CA, USA).

### 2.4 Thiobarbituric acid reactive substances (TBARS) assay

To measure the levels of oxidative stress, a TBARS assay was performed using the method described by Hu et al. (1989). Briefly, egg homogenates were added to two volumes of 7.5% trichloroacetic acid and mixed. The mixture was centrifuged at 1000 × *g* for 10 min. A supernatant was added to half the volume of 0.7% 2-thiobarbituric acid and boiled for 10 min. The mixture was cooled, and TBARS were measured at OD_532 nm_. The extinction coefficient of 156,000 M^−1^cm^−1^ was used for calculation.

### 2.5 Iron assay

To measure the ferrous and ferric iron concentrations of the egg homogenates, a Quantichrome™ iron assay kit (BioAssay Systems, Hayward, CA, USA) was used following the manufacturer's protocol for a 96-well plate.

### 2.6 Embryonic fixation and scaling

**Figure 1:**
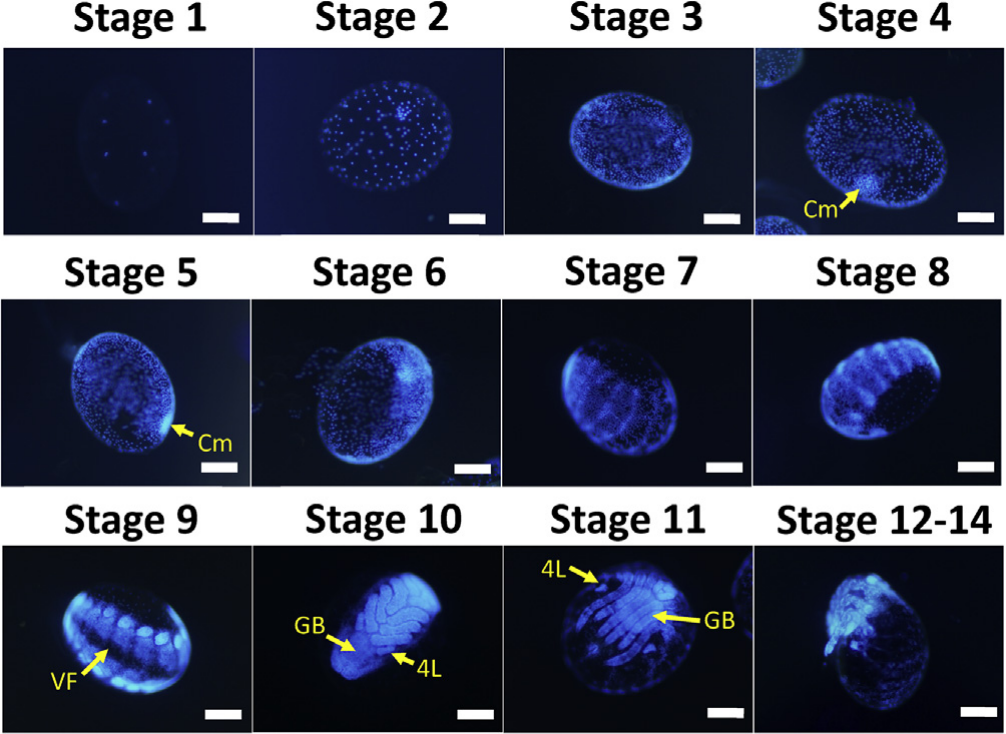
Stages of the embryonic development of *H. longicornis*. Embryos were deshelled and scaled. The embryonic nuclei were stained with DAPI and observed under fluorescence microscopy. The embryos were staged based on the classification system by Santos et al. (2013). Cm: cumulus cells; VF: ventral furrow; GB: germ band; 4L: 4^th^ set of limbs. Scale bars = 200 μm.

For embryonic fixation and scaling, the protocol by Santos et al. (2013) was followed with some modifications. Briefly, approximately 100 mg of eggs were separated for fixation and placed in a 1.5 ml tube. The eggs were washed for 8 min with a solution containing 1.5% sodium hypochlorite and 5% sodium carbonate. Subsequently, the eggs were washed with Milli-Q water three times. One milliliter of Milli-Q water was added to the tube, which was then heated at 90 °C for 2 min using a heat block. Immediately following this heating procedure, the tube containing the eggs was left on ice for 2 min, causing the cracking of the eggshells. Shortly thereafter, a fixation solution containing heptane and 4% paraformaldehyde in PBS (1:1) was added. The eggs remained suspended between the phases of the fixation solution, which was rotated at room temperature for 1 h. The lower phase containing the paraformaldehyde was removed, 100% methanol was added to the solution, and the tube was vigorously shaken for 2 min. Eggs that had lost their shells sank toward the bottom of the tube, whereas eggs that retained their shells remained at the interface between the phases of the solution. The eggs were washed three times with methanol, then washed three times with PBS. One milliliter of 20% sucrose solution was added and put in a shaker at 4 °C for 24–48 h. After removing the sucrose solution, 1 ml of SCALEVIEW A-2 (Olympus, Tokyo, Japan) was added and put in a shaker at 4 °C for 7 days. The eggs were then observed under a fluorescence microscope and classified based on the system by Santos et al. (2013) (Figure 1).

### 2.7 Differential egg count during oviposition

Scaled eggs during the different days of oviposition were classified. One hundred random eggs were observed and tallied based on the classification system by Santos et al. (2013). Since it is difficult to distinguish between stages 12–14 of the embryo during counting, these stages were grouped as one.

### 2.8 Statistical analysis

Statistical analyses were performed using STATA 15.0 software. Since the data is not normal and the variances are not equal, a Kruskal–Wallis rank test with Bonferroni multiple comparison tests was applied. Statistical significance was set as ^∗^*P <* 0.05.

## 3 Results

### 3.1 Transcription profiles of *GSTs* and *Fers* genes during embryogenesis

**Figure 2:**
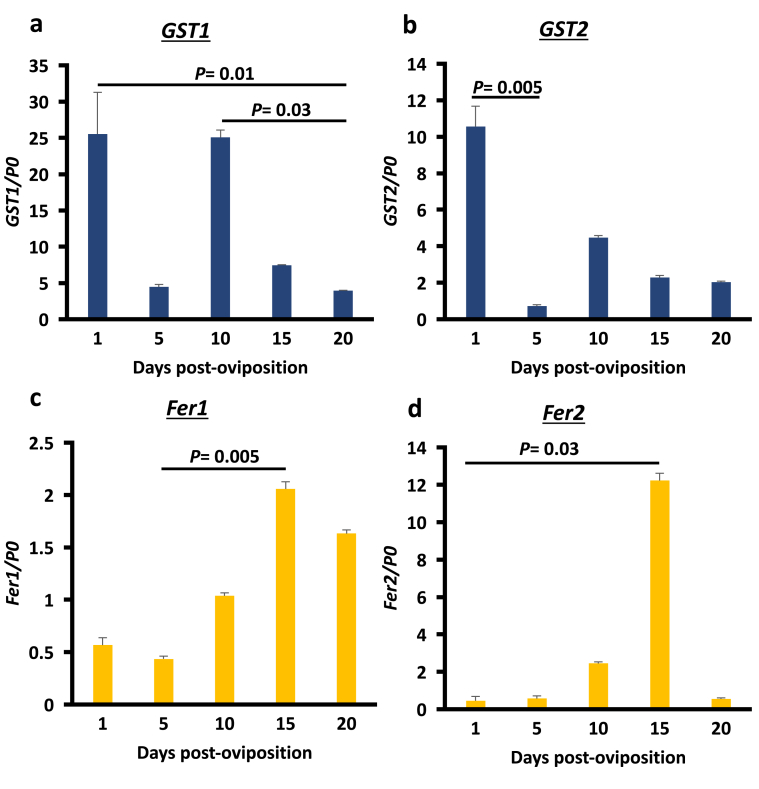
Transcription profiles of (a) *GST1*, (b) *GST2*, (c) *Fer1*, and (d) *Fer2* genes during *H. longicornis* embryogenesis. Total RNA was prepared from embryos harvested at different days post-oviposition. *P0* genes were used as the control. Error bar represents the mean ± standard deviation. Significant difference was determined using the Kruskal–Wallis rank test.

RT-qPCR was performed to analyze the transcription profiles of *GST1*, *GST2*, *Fer1*, and *Fer2* in different embryonic stages (Figure 2). *GST1* and *GST2* genes showed the highest expression levels during day 1 post-oviposition. At day 5 post-oviposition, the expression of the *GSTs* decreased, especially *GST2*, which decreased significantly (*P=*0.005). This was followed by an increase on day 10. After day 10 post-oviposition, both *GST1* and *GST2* genes decreased in expression and continued to decrease at day 20 post-oviposition, wherein the *GST1* was significantly decreased at day 20 in comparison to day 1 (*P=*0.01) and day 10 (*P=*0.03). Conversely, *Fer1* and *Fer2* gene expression significantly increased from day 1 to day 15 (*P=*0.005 and *P=*0.03, respectively), followed by decreased expression at day 20 post-oviposition. These results indicate that embryogenesis affects the transcription profiles of oxidative stress–related genes, including *GSTs* and *Fers.*

### 3.2 Protein expression profiles GSTs and ferritins during embryogenesis

**Figure 3:**
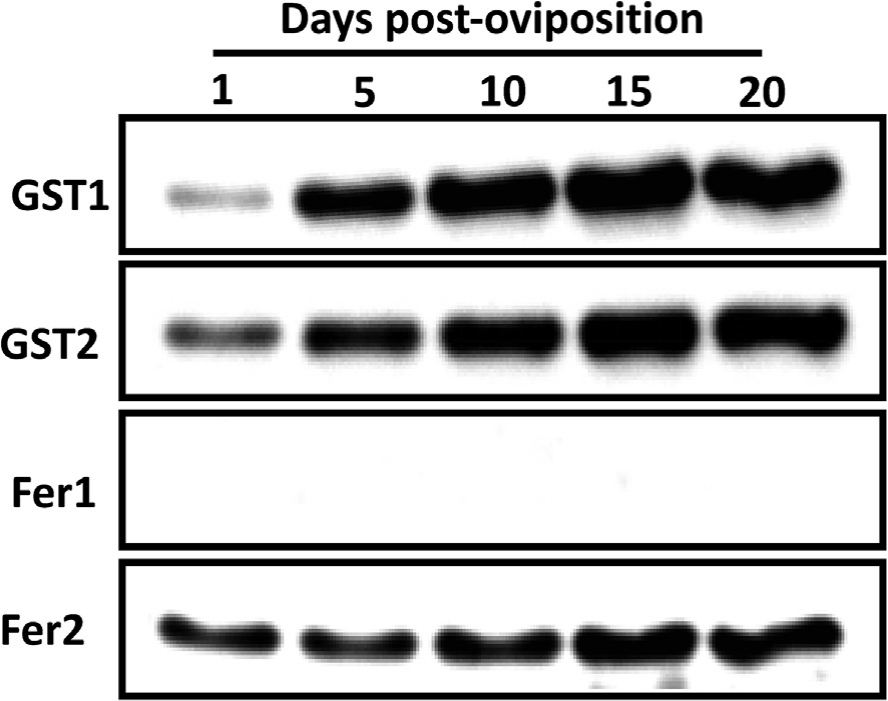
Expression profiles of GSTs and ferritins during *H. longicornis* embryogenesis. Proteins were prepared at different days post-oviposition. The results of Western blotting are shown as representative data of three separate experiments showing the same trend. The protein concentration was determined using a Micro BCA kit and maintained at 50 μg per lane before loading for Western blotting. Full blot images are available as a supplementary material (Figs. S1–S4).

Western blot analysis was performed to detect GST1, GST2, Fer1, and Fer2 during embryonic development (Figure 3). Full blot images are available as a supplemental material (Figs. S1–S4). Both GST1 and GST2 protein concentrations increased during the embryogenesis. Conversely, Fer2 protein expression appeared to be constitutively expressed from days 1–20 post-oviposition. On the other hand, the Fer1 protein was not detected in any developmental stages. These results show that embryogenesis affects the GST and ferritin protein expressions. The GST proteins appear to be transcriptionally regulated; however, the ferritin proteins are not.

### 3.3 Malondialdehyde concentration of embryos at different stages

**Figure 4:**
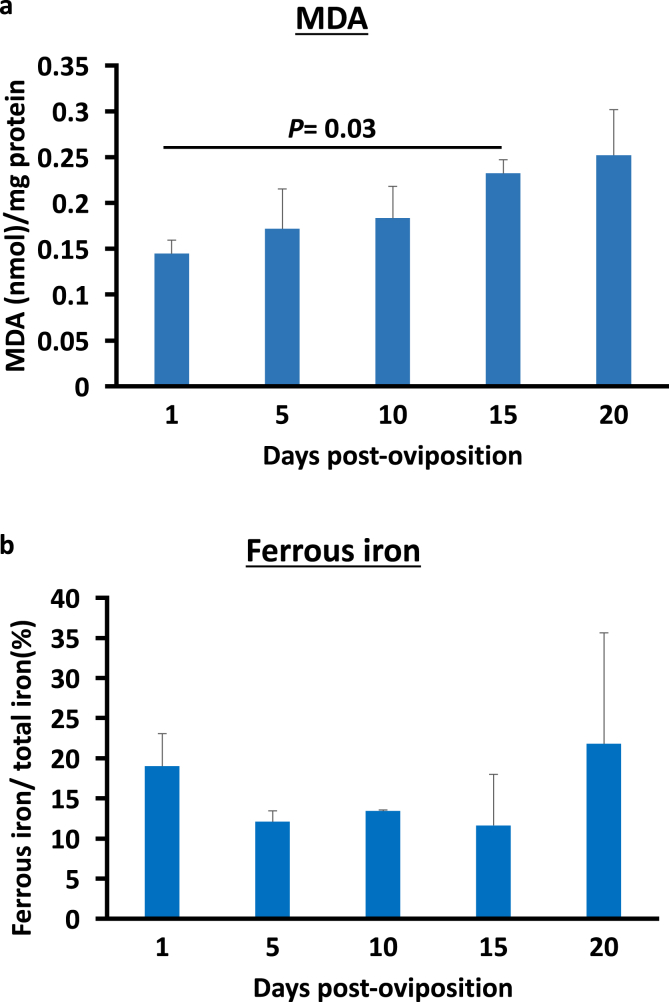
(a) Malondialdehyde concentrations during the embryogenesis of *H. longicornis.* MDA concentration was determined by TBARS assay. Significant difference was determined using the Kruskal–Wallis rank test. (b) Ferrous iron percentages during *H. longicornis* embryogenesis. The total iron concentration was determined using an iron assay kit, while the ferrous iron concentration was determined using the same kit without a reducing agent. The ferrous iron percentage is the concentration of ferrous iron divided by the total iron concentration.

As GSTs and ferritins are related to oxidative stress, a TBARS assay was performed to measure the levels of oxidative stress by measuring the MDA concentration at different embryonic stages (Figure 4a). The levels of MDA appeared to increase during the course of development, with a significant increase from day 1 to day 15 post-oviposition (*P=*0.03). This result indicates that embryonic development entails oxidative stress.

### 3.4 Ferrous iron concentration of embryos during embryogenesis

Since ferritins are among the major molecules used to regulate the ferrous iron concentration in ticks, an iron assay was performed to measure the ferrous iron concentration during the course of embryogenesis (Figure 4b). The ferrous iron concentration in the eggs appeared to be maintained from days 1–20 post-oviposition, as no significant change in ferrous iron concentration was observed. These results suggest that the tick embryos have a mechanism to maintain the ferrous iron concentration during development.

### 3.5 Developmental staging system of the embryo

The ratio of each developmental stage at each time point was determined via DAPI staining on the embryo after its fixation and scaling; it was observed under a fluorescence microscope to determine the events occurring in the embryo during that specific time point (Table 2). At day 1 post-oviposition, 98% of the eggs belonged to stage 1, wherein the embryo has been cellularized. At day 5 post-oviposition, 1–21% of the eggs belonged to stage 2, 16–34% to stage 3, 34–51% to stage 4, and 9–20% to stage 5. In stages 2 and 3, cellular proliferation intensifies, while in stages 4 and 5, the formation and migration of cumulus cells take place (Figure 1). At day 10 post-oviposition, 4–39% of the eggs belong to stage 9, in which the ventral furrow is formed (Figure 1). Also on the same day, 49–58% of the eggs are at stage 10 of development, wherein the ventral sulcus closes and the appendages increase in size (Figure 1). At this stage, a germ band was also identified (Figure 1). At day 15 post-oviposition, 80–98% of the eggs belong to stage 11, wherein there is a continuous increase in the limb buds and the germ band becomes shorter and broad (Figure 1). The fourth leg, as well as the ventral furrow, also starts to regress at this stage. Finally, at day 20 post-oviposition, 85–98% of the eggs belong to a combination of stages 12–14, wherein there is dorsal closure and the eggs are almost hatched (Figure 1). These results imply that DAPI staining, together with the scaling of eggs, could be a useful tool to visualize the events occurring during embryogenesis.

**Table 2:** Proportion of the different embryo stages at different days postoviposition (%).

DPO	Stages											
	1	2	3	4	5	6	7	8	9	10	11	12–14
1	98	1–2	1	0	0	0	0	0	0	0	0	0
5	0–1	1–21	16–34	34–51	9–20	1–12	0	0	0	0	0	0
10	0–1	0–1	1–18	0–9	0–5	2–9	0–2	0–5	4–39	45–58	0–2	0
15	0	0	0	0	0	0	0	1–5	0–11	1–3	80–98	0–2
20	0–1	0–1	1–4	0–1	0	1–2	0	1–3	0–1	1–2	0–2	85–93

DPO: days postoviposition.

## 4 Discussion

Embryogenesis refers to the period of development from fertilization to the differentiation of tissues. Embryogenesis is a product of well-controlled molecular and cellular-programmed proliferation and differentiation of cells (Ufer and Wang, 2011). This complex process of development requires energy from adenosine triphosphate (ATP) through glycolysis or oxidative phosphorylation. In the hard tick *Rhipicephalus microplus*, the same energy demand was observed during embryogenesis (Campos et al., 2007, 2011).

The female *H. longicornis*, after the completion of feeding, detaches from the host and initiates oviposition afterward. In previous literature, hatching was observed beginning on day 17 after egg laying (Shiraishi et al., 1990), while during the course of this study, hatching commenced from around day 20 post-oviposition. The phospholipoglycoprotein in the yolk or the vitellin, acquired during oogenesis as vitellogenin, would be an energy source during embryogenesis through mitochondrial respiration and oxidative phosphorylation (Raikhel and Dhadialla, 1992). However, the use of oxygen as part of the substrate for energy poses a potential hazard, as it could result in the production of ROS and may cause the modification of several macromolecules, some of which are lipids, proteins, DNA, and RNA. Therefore, embryonic cells must have a mechanism to protect the cells from ROS and oxidative stress (Ufer and Wang, 2011). In *R. microplus*, an increasing oxygen consumption was observed during embryogenesis (Freitas et al., 2007). In this study, increasing levels of MDA, which is an indicator of lipid peroxidation, were observed from day 1 to day 15 post-oviposition (Figure 4a). In *R. microplus* eggs, increasing levels of MDA were also observed during the progression of embryogenesis (Freitas et al., 2007). This could further indicate that embryogenesis results in oxidative stress in ticks, and ticks possess a mechanism to maintain the redox balance in order to survive.

In ticks, maintenance of the redox balance is achieved through a complex antioxidant system composed mostly of enzymes (Budachetri and Karim, 2015; Kumar et al., 2016; Kusakisako et al., 2018). Included in the plethora of enzymes are the GSTs. The utilization of GSTs during periods of oxidative stress, such as blood feeding, was expressed by the increased gene and/or protein expression of these molecules (Dreher-Lesnick et al., 2006; Perner et al., 2016; Hernandez et al., 2018b).

During embryogenesis, *R. microplus* GST activity was observed to increase from day 1 to day 20 after oviposition (Freitas et al., 2007). In our study, both GST1 and GST2 proteins increased as embryogenesis occurred from day 1 to day 20 (Figure 3). On the other hand, the *GST1* and *GST2* gene expression appeared to peak at day 1 and day 10 post-oviposition (Fig. 2a and b). The high *GST1* gene expression on day 1 post-oviposition could be derived from the deposited maternal mRNA. After fertilization, there is a period of transcriptional silence. During this period of quiescence, the embryonic genome in the nucleus is not yet expressed; therefore, the development occurring at this period, such as cellularization, is directed by cytoplasmic factors wherein the majority are maternal mRNA (Winata and Korzh, 2018). This could indicate the crucial role of GSTs during the early stages of development, as maternally derived mRNAs are molecules important to early embryonic development (Dworkin and Dworkin-Rastl, 1990). The decrease in the expression of *GST* and *GST2* genes at day 5, wherein continuous cellularization is occurring, could be attributed to the gradual decrease of the maternal mRNA in this period. Upregulated *GST* genes were observed on day 10. During this stage, the germ band is already formed, with the distinction of each area. This indicates that cellular differentiation among organs could have already occurred. At day 9 post-oviposition in *Hyalomma dromedarii*, the tick embryo's basic protein is at its highest level. This indicates that the embryo during this stage has the necessary amount of protein or mRNA to synthesize new molecules on its own (Ibrahim, 1998). After the increase at day 10 post-oviposition, a decrease in *GST* gene expression was observed from day 15 to day 20. Interestingly, the same expression pattern in zebra fish was observed in its mu-class GSTs (the same class of GST and GST2), wherein there is an increase in *GST* gene expression during the initial stages followed by decreasing *GST* gene expression in the later stages of development (Tierbach et al., 2018). In a study by Campos et al. (2007), mitochondrial exopolyphosphatase (PPX) activity in *R. microplus* was correlated to energy production in the mitochondria in embryos. An increase in PPX activity was shown after the day 4 oviposition, and the increase continued until its peak at day 7; it then decreased during days 12–18 of development. Therefore, oxidative phosphorylation increased during this period. This could also indicate an increase in oxidative stress from the redox reaction. This could trigger the transcription of these GSTs (Hayes and Pulford, 1995). It is also possible that another closely related *GST* gene that is more suitable to the events occurring at day 15 and day 20 after oviposition is favorably expressed. This is the period when distinct organs and features of the ticks can already be identified and the embryos are in preparation for hatching, since *Ixodes scapularis* has more than 20 identified GST transcripts (Reddy et al., 2011). The downregulation of a GST gene in favor of another GST gene has also been observed in the locust during chlorpyrifos exposure (Qin et al., 2014).

Although our study indicates the possible roles of GST1 and GST2 during embryogenesis, previous studies have shown that when *GST* genes were knocked down in eggs, no significant difference was observed in terms of hatching in comparison with the control group (Hernandez et al., 2018b). This indicates the capability of other GSTs to functionally compensate for the loss of another closely related GST. One example of this is the functional compensation of *GSTM2* when the *GSTM1* gene was knocked down in HeLa cells (Bhattacharjee et al., 2013).

Aside from mRNA and yolk nutrients that are maternally derived, other nutrients, such as iron, are acquired by the embryo from the adult female tick. Iron is transported to the embryo using iron-binding proteins such as ferritins and transferrins (Mori et al., 2014; Whiten et al., 2017). In this study, we checked the expression levels of the identified ferritins (*Fer1* and *Fer2*) in *H. longicornis*. Both *ferritins* have a low mRNA expression at the initial stage of embryogenesis (day 1 and day 5) and were upregulated from day 10 to day 15 and downregulated at day 20 (Fig. 2c and d). This further strengthens the hypothesis that the embryo-derived mRNA is expressed abundantly beginning on day 10 post-oviposition. This could also be an indication of organogenesis during this period, especially at day 15, when the expression of ferritin genes, especially *Fer2*, is highest. Incidentally, the organogenesis in *Hy. dromedarii* also occurs at day 15 post-oviposition (Ibrahim, 1998). In the study of Galay et al. (2013), the Fer2 is sourced from the midgut and secreted as a protein into the hemolymph, wherein it would be transported to other organs, including the ovaries and oocytes. This is further supported by Western blotting results, wherein, even with the very low *Fer2* gene expression at days 1–5 post-oviposition, the Fer2 protein is still constantly expressed throughout the embryogenesis (Figure 3). This could indicate that the Fer2 protein detected at the earlier stages, before organogenesis, such as from day 1 to day 5, is the maternally derived Fer2 that was taken in by the ovary and oocytes. No Fer1 protein was detected, even with the upregulation of gene expression at day 10 and day 15 (Figure 3). This could be because the intracellular ferritin expression, including that of Fer1, in tick cells is regulated by the interaction of iron-responsive element (IRE) in the *ferritin* mRNA with an iron-regulatory protein (IRP). On the other hand, IRP activity is dependent on the ferrous iron concentration in its environment (Hernandez et al., 2018c). Since no drastic change in the concentration of ferrous iron was observed during the embryogenesis, there is no induction of the protein expression of Fer1.

The nondetection of a Fer1 protein and the consistency of expression of Fer2 indicate that Fer2 could be the dominant protein that helps to maintain iron homeostasis in the embryo. It is also to be noted that the other iron-shuttling protein, transferrin, can be detected in the ovary but not in the eggs (Mori et al., 2014). This could further support the importance of Fer2 in embryogenesis. The knockdown of Fer2 in adult females, which also resulted in the lack of Fer2 in the eggs, caused abnormal morphology and lower hatchability in eggs (Galay et al., 2013). In *Drosophila*, the mutation of ferritins also resulted in abnormalities and death in embryos due to ectopic apoptotic events (González-Morales et al., 2015). ROS are known to be mediators of apoptosis, and the ferrous iron, through a Fenton reaction, could trigger the production of these ROS (Galay et al., 2014b, 2015). Therefore, the absence of functional ferritin could have resulted in increased ferrous iron that may result in these abnormal apoptotic events.

## 5 Conclusions

Embryogenesis is the only developmental stage in the tick that does not require blood meal. Nevertheless, the processes involved in this stage, including energy production and iron metabolism, would still result in the formation of ROS that could lead to oxidative stress. In our study, we showed that ticks could be utilizing oxidative stress–related molecules that were previously indicated during blood feeding, such as GSTs and Fer2. This is manifested by the increasing or constant expression of these molecules during the course of embryogenesis. This is further supported by the effect on egg fertility in knockdown and vaccination studies with these molecules. After vaccination with recombinant GST1, animals infested with *R*. *microplus* showed a 10% reduction in egg fertility (Oldiges et al., 2016). In *R. appendiculatus*, a 33% reduction in egg fertility was reported (Sabadin et al., 2017). On the other hand, the knockdown of Fer2 resulted in an 89% reduction in egg hatching in the *H. longicornis* tick, while vaccination experiments on it resulted in a 20% decrease in egg hatching and a 49% reduction in larval survival (Galay et al., 2013, 2014a). In *Ixodes ricinus*, vaccination with Fer2 resulted in an 85% reduction in egg fertility, while a 40% reduction was observed in *R. microplus* and a 51% reduction in *R. annulatus* (Hajdusek et al., 2010). This study, along with the previous studies, shows that the oxidative stress–related molecules GST and Fer2 could play a vital role in the embryogenesis of *H. longicornis* ticks.

## Declarations

### Author contribution statement

E. Hernandez: Conceived and designed the experiments; Performed the experiments; Analyzed and interpreted the data; Wrote the paper.

K. Shimazaki: Conceived and designed the experiments; Performed the experiments; Analyzed and interpreted the data.

T. Tanaka: Conceived and designed the experiments; Analyzed and interpreted the data; Contributed reagents, materials, analysis tools or data.

H. Niihara: Performed the experiments.

R. Umemiya-Shirafuji: Analyzed and interpreted the data; Contributed reagents, materials, analysis tools or data.

K. Fujisaki: Analyzed and interpreted the data.

### Funding statement

This work was supported by the Japan Society for the Promotion of Scienhce (JSPS) KAKENHI Grant Numbers 16H05028 and 17K19328, a Cooperative Research Grant (2019 joint-8) from the National Research Center for Protozoan Diseases, Obihiro University of Agriculture and Veterinary Medicine, the Takeda Science Foundation, and the Kieikai Research Foundation. E. Hernandez was supported by the Japanese Government's Ministry of Education, Culture, Sports, Science and Technology Scholarship (Monbukagakusho: MEXT) for doctoral fellowship.

### Competing interest statement

The authors declare no conflict of interest.

### Additional information

No additional information is available for this paper.

## Appendix A Supplementary data

Full blot image Full blot image
