# Supplementary material for: Expression analysis of glutathione S-transferases and ferritins during the embryogenesis of the tick Haemaphysalis longicornis
Source: Heliyon. 2020 Mar 30;6(3):e03644. doi: 10.1016/j.heliyon.2020.e03644 (PMC7114739; doi:10.1016/j.heliyon.2020.e03644)
Supplement: Full blot image [file mmc1.pdf]

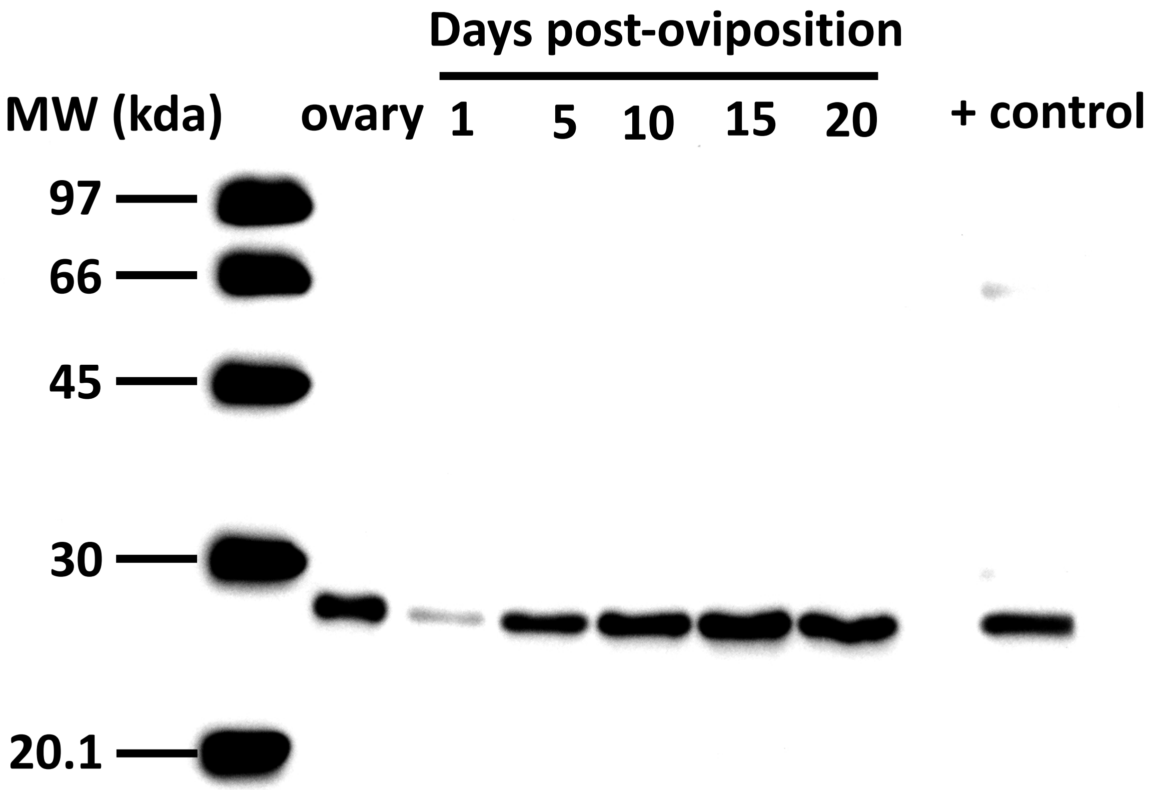

Figure S1. Full image of expression profiles of GST1 during *H. longicornis* embryogenesis. Proteins were prepared at different days postoviposition.. The protein concentration was determined using a Micro BCA kit and maintained at 50 μg per lane before loading for Western blotting.

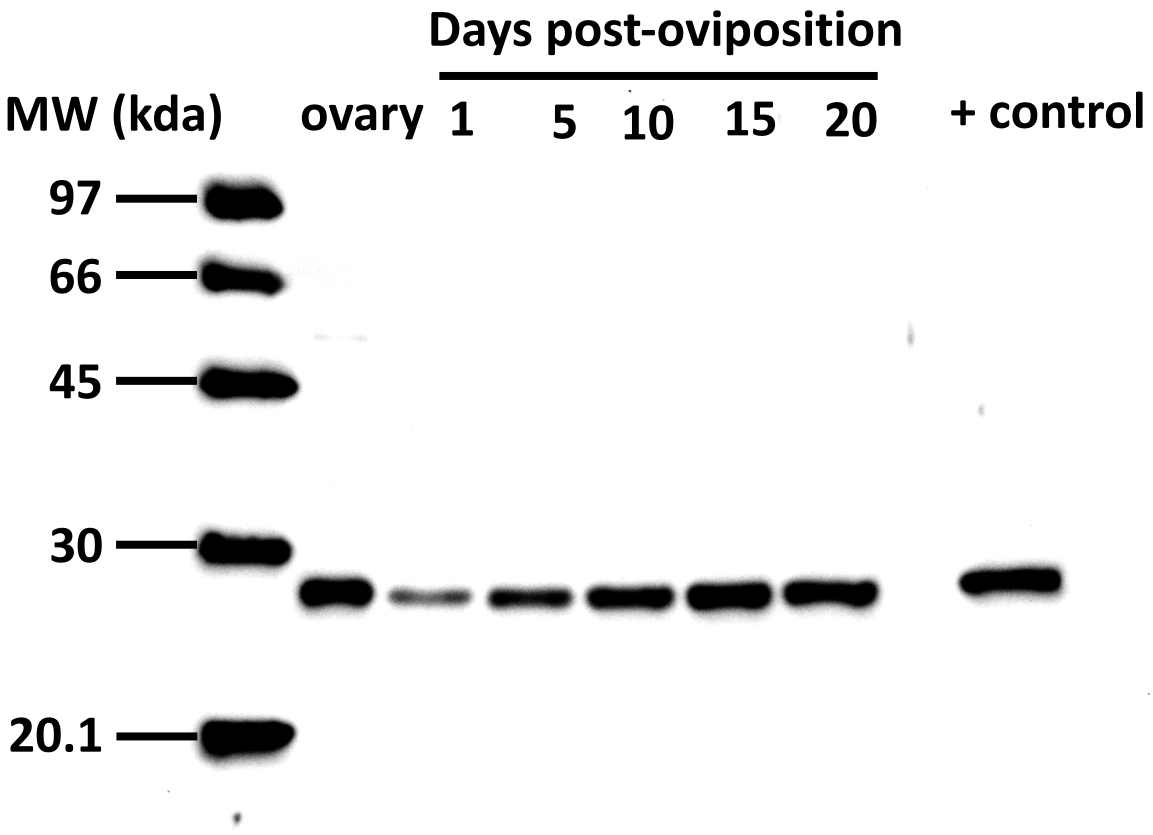

Figure S2. Full image of expression profiles of GST2 during *H. longicornis* embryogenesis. Proteins were prepared at different days postoviposition.. The protein concentration was determined using a Micro BCA kit and maintained at 50 μg per lane before loading for Western blotting.

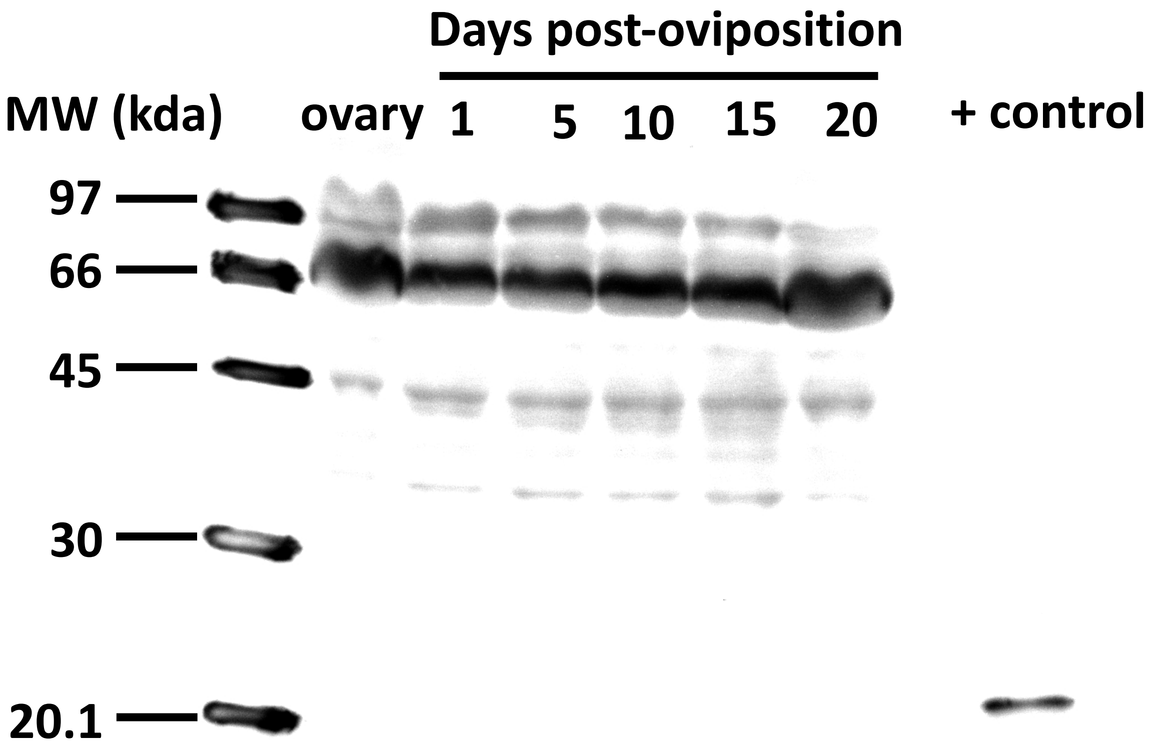

Figure S3. Full image of expression profiles of FER1 during *H. longicornis* embryogenesis. Proteins were prepared at different days postoviposition.. The protein concentration was determined using a Micro BCA kit and maintained at 50 μg per lane before loading for Western blotting.

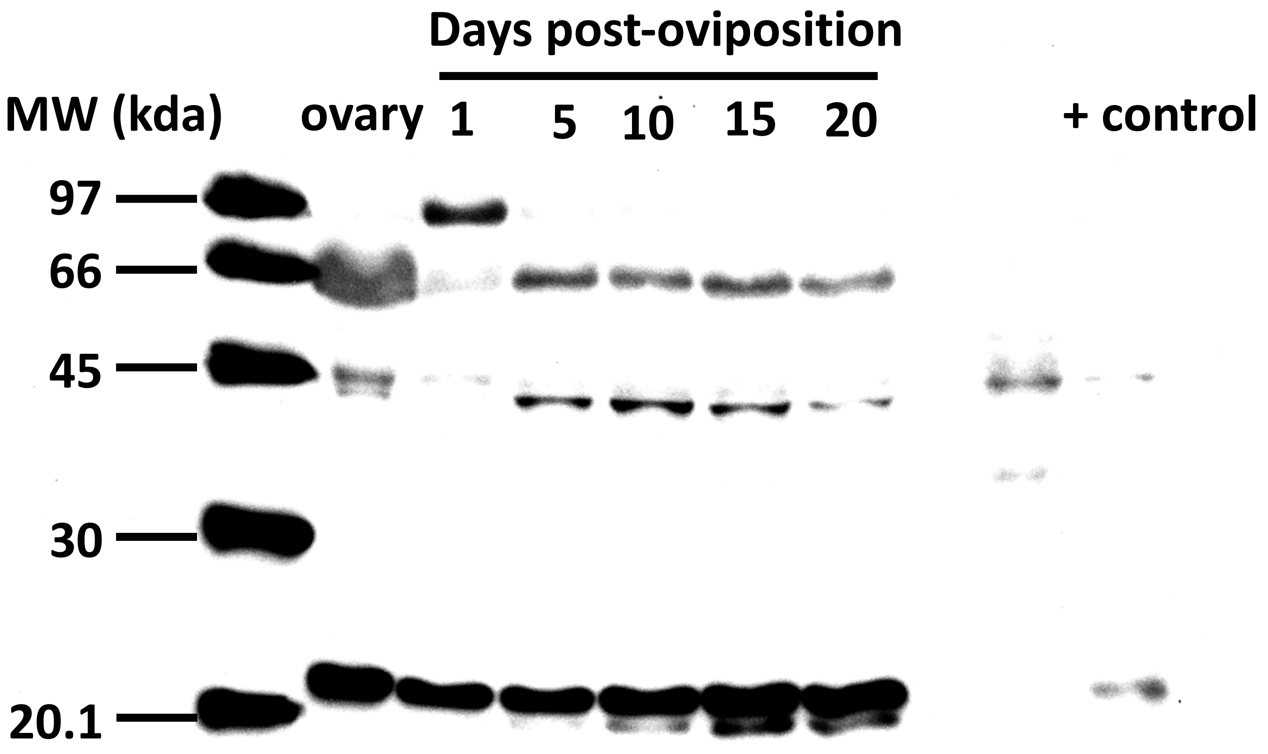

Figure S4. Full image of expression profiles of FER2 during *H. longicornis* embryogenesis. Proteins were prepared at different days postoviposition.. The protein concentration was determined using a Micro BCA kit and maintained at 50 μg per lane before loading for Western blotting.
